# Dietary Predictors of Paraben Exposure Among Adults in Northern Thailand

**DOI:** 10.3390/ijerph23050686

**Published:** 2026-05-21

**Authors:** Vivat Keawdounglek, Pussadee Laor, Warapon Paenkhokuard

**Affiliations:** Program of Environmental Health, School of Health Science, Mae Fah Luang University, Chiang Rai 57100, Thailand; pussadee.lao@mfu.ac.th (P.L.); 5931807016@lamduan.mfu.ac.th (W.P.)

**Keywords:** parabens, dietary exposure, processed foods, logistic regression, Thailand

## Abstract

**Highlights:**

**Public health relevance—How does this work relate to a public health issue?**

- Dietary exposure to parabens represents an under-recognized public health issue in rapidly changing food environments.
- This study links modern food consumption patterns with chemical exposure in a real-world population setting.

**Public health significance—Why is this work of significance to public health?**

- Identifying soft drinks as a consistent predictor provides a clear, actionable target for exposure reduction.
- The findings provide empirical evidence for prioritizing specific processed food categories in public health risk assessment and intervention strategies.

**Public health implications—What are the key implications or messages for practitioners, policy makers and/or researchers in public health?**

- Findings can inform population-level strategies to reduce preservative exposure through dietary modification.
- Policymakers and practitioners can use these results to design targeted interventions in similar food environments.

**Abstract:**

**Background**: Parabens are frequently utilized as preservatives in processed foods; nevertheless, the primary dietary factors contributing to exposure in northern Thailand remain undetermined. **Methods**: A cross-sectional study was conducted among 130 adults in Northern Thailand. Dietary intake was assessed using self-reported food consumption data combined with previously measured paraben concentrations. Due to the skewed distribution of intake, participants were classified into lower and higher exposure groups. LASSO regression was applied for variable selection, followed by multivariable logistic regression to identify dietary predictors of exposure. **Results**: Several processed food items were significantly associated with higher paraben exposure, including soft drinks, potato chips, and canned fish. No demographic factors were significantly associated with exposure. The final model demonstrated good explanatory power and classification performance. **Conclusions**: These findings suggest that routine consumption of certain processed foods and beverages may play a larger role in exposure than individual characteristics, and they highlight practical targets, particularly soft drinks, potato chips, and canned fish, for community-based health-promotion strategies aimed at reducing unnecessary preservative intake.

## 1. Introduction

Parabens have been widely used for decades as preservatives in a range of products, including beverages and processed foods. They were generally considered safe at low concentrations, which shaped earlier regulatory frameworks. However, human exposure to parabens occurs through dietary intake, inhalation, and dermal absorption, and has been widely documented in biomonitoring studies. Although these compounds are rapidly metabolized, concerns remain regarding their potential endocrine-disrupting effects and oxidative stress associated with repeated exposure [1,2]. With increasing evidence, attention has shifted toward understanding dietary exposure, particularly given the widespread consumption of processed and ready-to-drink foods. As these products have become part of routine dietary habits, diet represents an important route of exposure that warrants closer examination.

**Figure 1:**
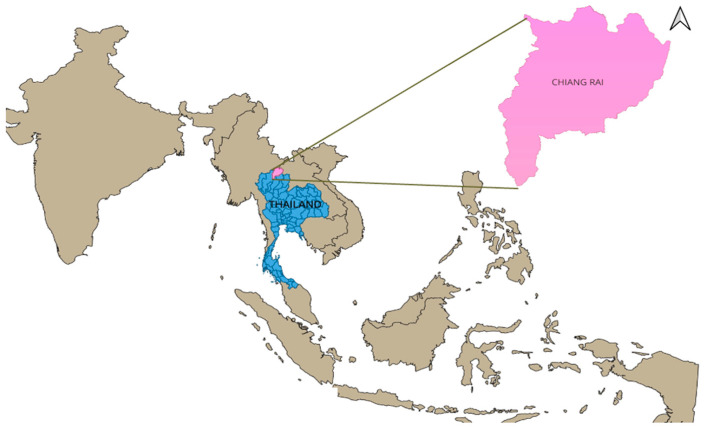
Study area: Chiang Rai Province, Thailand. The pink highlighted area represents Chiang Rai Province.

Asian research supports the concept. In Japan, the Philippines, Malaysia, and China [3,4,5,6], parabens have been identified in canned products, drinks, and other foods. These findings suggest that food exposure may encompass more than just a limited number of products and may indicate consumer purchasing and consumption patterns regarding packed meals. Thai studies discovered parabens in fruit juice, energy drinks, beer, and spirits; meanwhile, the use of parabens in food is regulated by the Ministry of Public Health, Thailand, with allowable levels varying by food category. In general, parabens are permitted at concentrations of up to 0.1% (1 g/kg) in certain products, such as canned vegetables and pickled foods, while lower limits (e.g., 0.05%) may apply to specific preserved food [7]. Methyl, ethyl, and propyl parabens were detected in the urine of preschoolers in Chiang Rai, indicating that common products may be a source [8]. Nonetheless, no Thai research has meticulously examined the dietary categories and demographic groups most significantly associated with elevated paraben consumption. Adults consume more ready-made foods than children because of their jobs, convenience, and routine life patterns [9]. A clear understanding of dietary patterns requires identifying the key food groups contributing to overall intake. People in northern Thailand, particularly in Chiang Rai Province (see Figure 1), may have distinct dietary patterns because of the local food preferences and the fact that they may purchase packaged foods from nearby borders [10].

Since individuals consume many types of foods at the same time, and some of these foods overlap in ingredients, it is useful to apply an analytical approach that can sort through multiple variables together. Penalized regression techniques such as the Least Absolute Shrinkage and Selection Operator (LASSO) allow researchers to identify the most relevant predictors without overfitting and are particularly suitable for studies that involve numerous correlated food variables [11,12,13]. Based on these considerations, the present study aims to identify demographic and food-related predictors associated with higher dietary intake of parabens among adults in northern Thailand. By applying LASSO for variable selection and following up with multivariable modelling, the analysis highlights which products and population groups contribute most to predicted intake. The findings are intended to support local public-health agencies in planning practical communication strategies and improving awareness of preservative-related dietary exposure in the region [14,15,16].

## 2. Materials and Methods

### 2.1. Conceptual Framework

**Figure 2:**
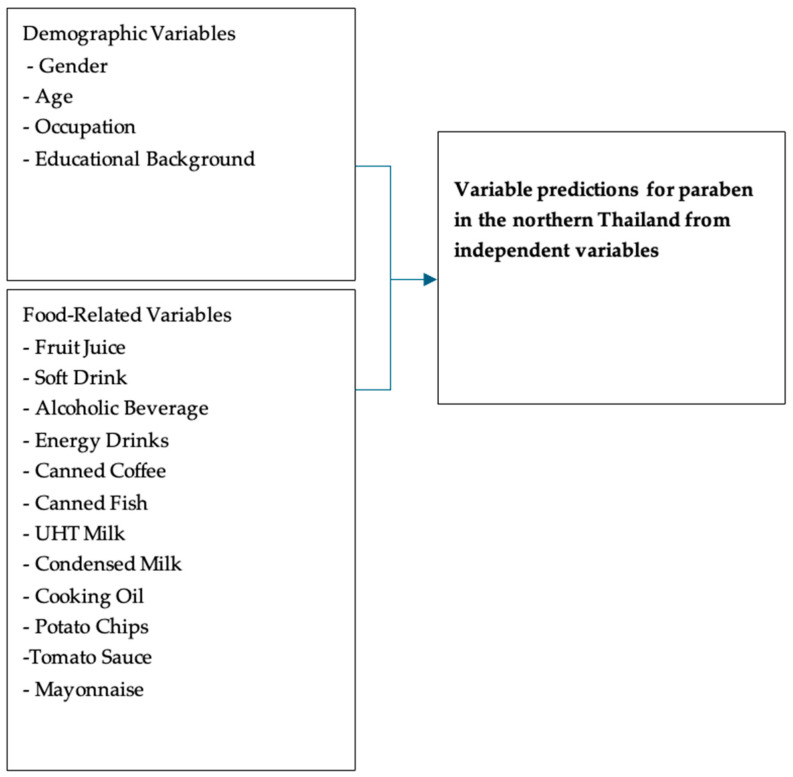
The independent and dependent variables of this study.

This cross-sectional survey was carried out in Chiang Rai Province, Thailand, to investigate potential dietary exposure of methylparaben (MeP), ethylparaben (EtP), propylparaben (PrP), isobutylparaben (iBuP), and butylparaben (BuP) [8,17,18]. Sample size was calculated using G*Power 3.1 for logistic regression (test family: Z tests, model: logistic regression) [19]. For comparison of two independent proportions (two-tailed test, α = 0.05, power = 0.80, expected odds ratio = 2.5, baseline probability p_0_ = 0.30, and predictor prevalence = 0.50) were used. G*Power indicates a required sample size of 59 per group (118 total). The per-group estimate follows the default structure used by G*Power for comparing two independent proportions. To allow for potential non-response and to increase study power, we recruited 130 participants. We hypothesized that demographic and food-related variables could predict dietary paraben intake at the 0.05 significance level. The conceptual framework consisted of demographic and food-related variables as independent predictors. Demographic variables included gender, age, occupation, and educational background. Food-related variables comprised consumption of fruit juices, soft drinks, alcoholic beverages, energy drinks, canned coffee, canned fish, UHT milk, condensed milk, cooking oil, potato chips, tomato sauce, and mayonnaise. The relationship between independent and dependent variables is illustrated in Figure 2.

### 2.2. Data Collection and Measurements

Data collection in this study was achieved by a self-administered questionnaire. A structured questionnaire was developed based on a review of the relevant literature (see Table S1) and consisted of the following sections [7,8,20,21,22,23,24,25,26]:

(1) Demographic information (gender, age, educational attainment, income, body weight, height, and calculated BMI);
(2) Consumption of ready-to-drink fruit juices (four brands);
(3) Consumption of soft drinks (four brands);
(4) Consumption of alcoholic beverages (two brands);
(5) Consumption of energy drinks (six brands);
(6) Consumption of canned coffee;
(7) Consumption of UHT milk (four brands);
(8) Consumption of condensed milk (five brands);
(9) Consumption of cooking oil;
(10) Consumption of canned fish (two brands);
(11) Consumption of potato chips;
(12) Consumption of tomato sauce;
(13) Consumption of mayonnaise.

Prior to data collection, the questionnaire underwent content validation using the IOC index and reliability assessment using Cronbach’s alpha [17,18]. The IOC value was 0.68 and Cronbach’s alpha was 0.95, indicating acceptable content validity and excellent internal consistency. Moreover, preliminary descriptive statistics (frequency and percentage) were analyzed using IBM SPSS version 30.0 (IBM Corp., Armonk, NY, USA) [27].

### 2.3. Dietary Paraben Estimation

Paraben concentrations in food products were determined using high-performance liquid chromatography (HPLC), as described in a related analytical study conducted by our research group [8,10]. The quantified concentrations (Table S2) were then combined with the participants reported dietary intake to estimate individual exposure, expressed as micrograms per kilogram body weight per day (µg/kg bw/day). Total daily intake of MeP, EtP, PrP, iBuP, and BuP was calculated following World Health Organization guidelines [28]

Dietary Exposure (µg/kg bw/day) = (C × IR)/BW(1)

where:

C is a concentration (µg/g)
IR is ingestion rate (g/day)
BW is body weight (kg)

For subsequent modeling, dietary paraben exposure was categorized into two groups (lower and higher exposure) based on the median (50th percentile) of the total exposure score. This binary classification approach was adopted to address the highly skewed distribution of the exposure data and to enable robust logistic regression analysis [29,30,31,32]. In addition, the paraben concentration data were derived from a related analytical study conducted in the same geographic area and during the same time as the dietary survey, ensuring consistency and representativeness of the exposure assessment.

### 2.4. Variable Selection and Predictive Modeling

Food variables were dichotomized into “ever consumed” versus “never consumed.” To reduce multicollinearity and identify relevant predictors among a large number of dietary variables, LASSO regression was performed using JASP software version 0.95.4 (The University of Amsterdam, Amsterdam, The Netherlands) [33,34]. The optimal penalty parameter (λ) was selected via 10-fold cross-validation, and variables with non-zero coefficients were retained for inclusion in the multivariable logistic regression model. Moreover, multicollinearity among the selected variables was assessed using variance inflation factors (VIFs), and all variables showed acceptable levels [35,36]. The selected predictors were subsequently entered into a multivariable binary logistic regression model using IBM SPSS Statistics version 30 (IBM Corp., Armonk, NY, USA) at a significance level of 0.05 [27,29,30,31,32]. Outputs included odds ratios (Exp(B)), *p*-values, and 95% confidence intervals. In addition, model performance was evaluated using Nagelkerke R^2^, classification accuracy, the Hosmer–Lemeshow (H-L) goodness-of-fit test, and the area under the receiver operating characteristic curve (AUC) [37,38].

## 3. Results

### 3.1. Participant Characteristics

According to the demographic characteristics of the participants (see Table S3), 79.2% were male, with an age range between 28 and 38 years. In terms of educational attainment, 93.1% had completed a bachelor’s degree or higher. The majority were employed as office workers (53.1%). Regarding health status, 54.6% of the participants had a body mass index (BMI) within the normal range.

### 3.2. Consumption Patterns of Paraben-Containing Foods

Table S4 shows that cooking oil was the most frequently consumed product, with 12 participants reporting daily intake. UHT milk brand A ranked second, with six individuals consuming it every day. In third place were UHT milk brand F, potato chips, and tomato sauce, all of which were consumed daily, with three respondents reporting daily intake.

For foods consumed 4 to 6 days per week, UHT milk brand F was the most common, with 22 participants reporting this frequency. Cooking oil followed, with 17 people indicating regular use within this range. In third place were potato chips and UHT milk brand A, each reported by 10 participants.

Among those who consumed the items only 1 to 2 days per week, cooking oil was again the most cited, with 47 participants falling into this category. UHT milk brand A came next, with 46 respondents. Interestingly, alcoholic beverage brand B appeared as the third most consumed item within the 1 to 2 days per week group.

To sum up, cooking oil and UHT milk were the most frequently consumed products.

### 3.3. Estimated Dietary Paraben Intake

**Figure 3:**
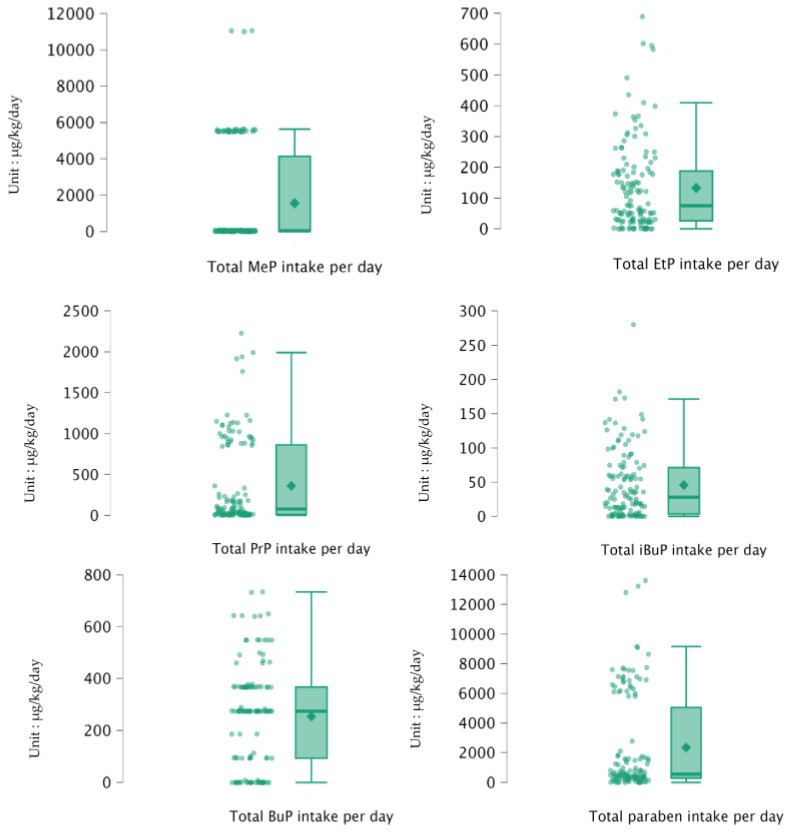
Maximum and average estimated total dietary paraben intake (µg/kg bw/day) among participants (*n* = 130). Error bars represent variability across food categories.

Dietary paraben intake varied widely among participants, and the distribution leaned heavily to the right. As shown in Figure 3, each food item contributed variably to the estimated exposure.

When broken down by individual compounds, participants were estimated to consume an average of 29.72 ± 27.69 µg/kg bw/day of total paraben. The distribution of estimated dietary paraben exposure was strongly right-skewed (skewness 1.297–5.074 across compounds); therefore, median values were reported to represent a central tendency. The median total paraben intake was 25.12 µg/kg bw/day (See Table S5). These wide ranges reflect substantial differences in food choices among individuals, which explains the highly skewed distribution and supports the use of binary exposure classification for subsequent analysis before running predictive models.

Table S6 displayed the consumption patterns of paraben-related food items. From this table, the consumption patterns varied noticeably across the different food categories. Several items were rarely consumed by the participants; for example, more than eighty-eight percent reported that they had never consumed canned coffee, UHT milk, or mayonnaise, and energy drinks were almost entirely absent from the diet of most respondents. In contrast, certain foods were more commonly used. Cooking oil, fruit juice, and potato chips showed higher levels of consumption, with the latter being the most frequently reported: nearly 74% of the participants indicated that they had eaten potato chips.

Due to the highly skewed distribution of dietary paraben intake, participants were categorized into lower (*n* = 62 or 47.7%) and higher exposure groups (*n* = 68 or 52.3%) for subsequent analysis.

### 3.4. LASSO-Selected Predictors with VIF Confirmation

As shown in Table 1, the LASSO procedure identified a small set of food-related variables as meaningful predictors of paraben exposure. Only variables with non-zero coefficients were retained for further analysis. In the final selection, three food-related variables, soft drink consumption, canned fish consumption, and potato chip consumption, were identified as relevant predictors. No demographic variables were selected by the LASSO procedure.

To assess potential multicollinearity among the selected predictors, variance inflation factors (VIFs) were examined. All VIF values were close to 1, indicating no evidence of multicollinearity among the retained variables. These results support the stability and independence of the selected predictors for subsequent logistic regression analysis.

### 3.5. Analysis of Binary Logistic Regression

Table 2 presents the results of the binary logistic regression analysis. Several processed food items were identified as significant predictors of higher dietary paraben exposure. Soft drink consumption was associated with increased odds of exposure (OR = 9.29, 95% CI: 3.34–25.82, *p* < 0.001), followed by canned fish (OR = 10.72, 95% CI: 3.43–33.48, *p* < 0.001) and potato chips (OR = 12.27, 95% CI: 3.13–48.09, *p* < 0.001).

The model demonstrated good performance, with a Nagelkerke R^2^ of 0.593 and an overall classification accuracy of 81.5%. The Hosmer–Lemeshow (H-L) test indicated a good model fit (*p* = 0.703), and the model showed strong discriminatory ability with an area under the ROC curve (AUC) of 0.884.

## 4. Discussion

Based on our findings, dietary paraben intake in this population exhibited a strongly right-skewed distribution. When dietary behavior patterns were examined, potato chips emerged as one of the most commonly consumed items, followed by fruit juice. After applying LASSO, VIF analysis, and binary logistic regression [39], several processed food items were identified as key predictors of paraben exposure. In particular, soft drink consumption showed the strongest and most consistent association with increased exposure. In addition, canned fish and potato chips were also significantly associated with higher paraben intake. These findings suggest that commonly consumed processed foods, particularly beverages and snack items, may play an important role in shaping dietary paraben exposure among northern Thai adults.

Our findings show a stronger association between paraben exposure and everyday eating patterns than with basic demographic characteristics such as age, gender, occupation, or BMI. This differs from findings reported by Kotb et al. [40] and Defta et al. [41], who observed gender differences in attitudes or purchasing choices related to food additives. These earlier studies focused on stated preferences, whereas our analysis is based on estimated exposure derived from self-reported dietary data. Reported behavior and actual intake do not always align, which may help explain the difference in outcomes.

In our analysis, demographic variables did not show a clear association with exposure. However, this finding should be interpreted with caution, as the relatively homogeneous study population may have limited variability in key characteristics, thereby reducing the statistical power to detect such relationships, and does not necessarily indicate the absence of true demographic effects. Moreover, differences in food availability, household eating routines, or how individuals interpret potential risks can vary across groups and may influence intake indirectly. A more complete understanding would likely require information that reflects local contexts more closely, for example, migration background [42], familiarity with guidance on reducing paraben intake [41,43], or the degree of social connectedness within a community [44]. Including elements of this kind may help explain variations that were not evident in the present analysis and support the development of health promotion efforts tailored to conditions in northern Thailand.

Soft drink consumption emerged as a strong predictor of paraben exposure in this study. Packaging for beverage products often interacts with liquids more than people typically anticipate. Plastic bottles, coated cans, and multilayer cartons all have components that can release small amounts of chemicals into the food under certain conditions. Liao et al. [5] and Chung et al. [45], for example, demonstrated that BPA can be detected in beverages due to container materials. Seref and Cufaoglu [46] also reported that parabens may originate from inks or coating layers rather than the food formulation itself. The migration process is complex and depends on factors such as storage duration, temperature, and the chemical properties of both the food and packaging materials. Liquids and semi-liquid products are particularly susceptible to this process, as they remain in continuous contact with the container surface. Therefore, part of the observed association between soft drink consumption and paraben exposure may reflect contributions from packaging materials, including films, adhesives, or coatings containing preservative-related compounds [46,47,48].

However, this study could not distinguish between contributions from food composition and packaging, as detailed packaging information was not collected. Future research incorporating packaging characteristics or targeted migration analyses would be valuable for clarifying these mechanisms. In addition to soft drinks, other processed food items such as canned fish and potato chips were also associated with higher paraben exposure. These products often undergo industrial processing and packaging, which may increase the likelihood of preservative use or chemical migration during storage [46,47,49,50]. Previous studies, including Monteagudo et al. [51], have reported that methylparaben (MeP) can be a major contributor to paraben exposure in certain processed food products. This supports the notion that some foods may disproportionately contribute to overall exposure, particularly when consumed frequently. In line with these findings, the present study suggests that commonly consumed processed foods may play an important role in shaping dietary paraben exposure among northern Thai adults [52,53].

In summary, soft drinks emerged as a consistent and significant predictor of dietary paraben exposure. Other processed food items, including canned fish and potato chips, were also associated with higher exposure levels. These findings suggest that commonly consumed processed foods may contribute to paraben intake through multiple pathways, including food formulation and possible packaging-related migration; however, this mechanism was not directly evaluated in the present study and should be interpreted with caution. Because detailed information on packaging characteristics and certain contextual factors was not collected, the contribution of packaging-related migration to the estimated exposure could not be determined. Future studies examining both food products and their handling practices in northern Thailand may help identify practical approaches to reduce exposure. Moreover, locally appropriate strategies to reduce the consumption of highly processed foods, particularly soft drinks, canned fish, and potato chips should be considered to help limit excessive paraben intake in this population.

However, this study has several limitations. First, the study population was relatively homogeneous and may not be fully representative of the general adult population, which could limit the generalizability of the findings [54]. Second, dietary intake was assessed using self-reported data, which may be subject to recall bias, social desirability bias, or reporting inaccuracies. Third, the relatively large odds ratios and wide confidence intervals observed in this study may reflect the modest sample size and the limited number of participants in certain exposure categories [55]. In addition, the dichotomization of both exposure and dietary variables may have reduced variability and contributed to less precise estimates. Therefore, the results should be interpreted with caution, particularly regarding the magnitude and precision of the observed associations [56]. Future studies with larger and more diverse samples are warranted to improve the robustness and generalizability of the findings.

## 5. Conclusions

This study demonstrates that typical dietary habits play a key role in shaping paraben exposure among northern Thai adults. Soft drink consumption emerged as the most consistent predictor of increased exposure following LASSO-based variable selection and multivariable logistic regression. Other processed food items, including canned fish and potato chips, were also significantly associated with higher exposure levels.

For this study, no significant differences were observed across demographic groups, suggesting that habitual dietary behaviors, rather than individual characteristics such as age, gender, occupation, or BMI, are the primary drivers of exposure in this population.

These findings highlight important opportunities for public health intervention. Reducing the consumption of highly processed foods, particularly soft drinks, and promoting safer food handling and packaging practices may help lower dietary paraben exposure. Future research incorporating packaging characteristics and local food practices would provide further insight into exposure pathways and support the development of targeted, context-specific strategies for northern Thailand.

## Figures and Tables

**Table 1:** Variable Selection for Paraben Exposure using LASSO and VIF.

**Variable**	**LASSO Coefficients** **(λ-penalized β)**	**Selected by LASSO (Yes/No)**	**VIF**
Demographic Variables			
Gender	0.000	No	
Age	0.000	No	
Education	0.000	No	
Occupation	0.000	No	
BMI	0.000	No	
Food Variables			
Fruit Juice	0.000	No	
Soft Drinks	0.105	Yes	1.140 *
Alcohol Beverages	0.000	No	
Energy Drinks	0.000	No	
Canned Coffee	0.000	No	
Canned Fish	0.100	Yes	1.122 *
UHT Milk	0.000	No	
Condensed Milk	0.000	No	
Cooking Oil	0.000	No	
Potato Chips	0.108	Yes	1.052 *
Tomato Sauce	0.000	No	
Mayonnaise	0.000	No	
**Summary of Selected Variables**	3 Food Variables (Soft Drinks, Canned Fish, and Potato Chips)		

* VIF values were computed only for predictors with non-zero LASSO coefficients. All VIF values were below 2, indicating no multicollinearity.

**Table 2:** Binary logistic regression results.

Predictor Variable	Model Analysis by Binary Logistic Regression		
	Exp (B)	*p*-Value	95% CI
Food predictors			
Soft Drinks	9.288	<0.001	3.341–25.821
Canned Fish	10.722	<0.001	3.434–33.479
Potato Chips	12.270	<0.001	3.131–48.092
Model Performance			
Nagelkerke R^2^	0.593		
Classification Accuracy (%)	81.5		
AUC	0.884		
H-L *p*-Value	0.703		

## Data Availability

The original contributions presented in this study are included in the article/Supplementary Material. Further inquiries can be directed to the corresponding author.

## Supplementary Materials

The following supporting information can be downloaded at: https://www.mdpi.com/article/10.3390/ijerph23050686/s1, Table S1: Questionnaire for the study of “Consumer Behavior and Health Risk Perceptions Related to Paraben Intake from Food Products in Northern of Thailand”; Table S2: Concentration of paraben in the food production; Table S3: The frequency and percentage of demographic information; Table S4: Consumption Behavior to intake food contaminated with paraben; Table S5: Descriptive Statistics for dietary paraben intake; Table S6: The consumption patterns of paraben-related food items.
