# Supplementary material for: Dietary Predictors of Paraben Exposure Among Adults in Northern Thailand
Source: Int J Environ Res Public Health. 2026 May 21;23(5):686. doi: 10.3390/ijerph23050686 (PMC13206236; doi:10.3390/ijerph23050686)
Supplement: Supplementary file 1 [file ijerph-23-00686-s001.zip › ijerph-4286228-supplementary.pdf]

Supplementary Material

S1 Table. Questionnaire for the study of "Consumer Behavior and Health Risk Perceptions Related to Paraben Intake from Food Products in Northern of Thailand"

Direction. Please mark X into the blank that related to your information

Part I Demographic information

Gender

___Male
___Female

Age

___Less than 28 years
___28-38 years
___39-48 years
___49-58 years

Educational attainment

__High school
__Diploma
__Bechelor'degree or higher

Occupation

__Government officer
__Office employee
__Academic personnel (e.g., teachers, lecturers)
__Students
__Self-employed

Monthly income

__Less than 15,000 THB
__15,000-30,000 THB
__More than 30,000 THB

Part II Food production intake

Direction. Please mark X into the blank that related to your daily life behavior

**Type of food**	**Frequency of consumption**			
	**Never**	**1-3 Day/week**	**4-6 Day/week**	**Everyday**
**Ready-to-drink fruit juices**				
A				
B				
C				
D				
**Soft Drink**				
A				
B				
C				
D				
A				
B				
**Energy drink**				
A				
B				
C				
D				
E				
F				
**Ready-to-drink canned coffee**				
A				
**UHT milk**				
A				
B				
C				
D				
E				
F				
**Condensed milk**				
A				

Type of food	Frequency of consumption			
	**Never**	**1-3 Day/week**	**4-6 Day/week**	**Everyday**
B				
C				
D				
E				
**Cooking Oil**				
A				
**Canned fish**				
A				
B				
**Potato chips**				
A				
**Tomato sauce**				
A				
**Mayonnaise**				
A				

Notice: For the article publication, we cannot publish the trademark due to the recommendation of MFU human ethical committee

Supplementary Material

S2 Table. Concentration of paraben in the food production

Type of Food	Concentration of paraben (µg/g)				
	Methyl Paraben	Ethyl Paraben	Propyl Paraben	Iso-butyl Paraben	Butyl Paraben
**Ready-to-drink fruit juices**					
A	2.643	ND	3.974	ND	ND
B	1.542	ND	3.636	ND	ND
C	2.670	6.097	1.687	ND	ND
D	0.921	1.001	2.938	ND	2.108
**Soft Drink**					
A	ND	5.108	ND	ND	6.394
B	1.278	ND	1.659	ND	ND
C	ND	2.207	2.066	ND	ND
D	0.359	2.076	0.651	ND	ND
**Alcoholic beverage**					
A	ND	0.6571	1.058	ND	1.077
B	1.1648	5.0289	1.962	0.733	2.241
**Energy drink**					
A	1.677	ND	3.662	1.2896	ND
B	0.758	0.504	ND	ND	ND
C	ND	ND	ND	ND	ND
D	4.516	2.771	ND	ND	1.937
E	ND	8.732	1.721	1.0528	9.167
**Ready-to-drink canned coffee**					
A	2.251	ND	6.424	7.069	0.558
**UHT milk**					
A	1.124	0.746	1.314	ND	1.417
B	0.673	3.541	ND	ND	ND
C	0.626	ND	ND	ND	ND
D	0.941	0.279	0.399	ND	ND
F	0.304	ND	1.111	1.449	ND
G	0.329	ND	2.229	2.773	ND
**Condensed milk**					
A	0.307	0.371	0.417	1.716	ND
B	0.557	0.895	1.478	1.302	ND
C	0.366	0.482	0.703	4.762	ND
E	ND	0.552	0.671	5.757	ND
F	0.404	0.477	0.638	3.467	ND

**Type of Food**	**Concentration of paraben (µg/g)**				
	**Methyl Paraben**	**Ethyl Paraben**	**Propyl Paraben**	**Iso-butyl Paraben**	**Butyl Paraben**
**Cooking Oil**					
A	0.267	0.296	0.246	0.888	ND
**Canned fish**					
A	0.343	ND	1.426	1.998	ND
B	0.321	ND	1.598	1.973	ND
**Potato chips**					
A	0.826	0.579	14.525	40.91	0.361
**Tomato sauce**					
A	0.542	ND	27.179	ND	2.167
**Mayonnaise**					
A	0.852	4.497	3.813	ND	1.124

Supplementary Material

S3 Table. the frequency and percentage of demographic information

**Demographic Information**	**n=130**	
	**Frequency**	**%**
**Gender**		
Male	103	79.2
Female	27	20.8
**Age**		
Less than 28 years	40	30.8
28-38 years	42	32.3
39-48 years	38	29.2
49-58 years	10	7.7
**Educational attainment**		
High school	6	4.6
Diploma	3	2.3
Bechelor'degree or higher	121	93.1
**Occupation**		
Government officer	20	15.4
Office employee	69	53.1
Academic personnel (e.g., teachers, lecturers)	9	6.9
Students	17	13.1
Self-employed	15	11.5
**Monthly income**		
Less than 15,000 THB	15	11.5
15,000-30,000 THB	61	46.9
More than 30,000 THB	54	41.5
**Body Mass Index**		
Underweight	20	15.4
Normal weight	71	54.6
Overweight	25	19.2
Obesity	14	10.8

Supplementary Material

S4 Table. Consumption Behavior to intake the food contaminated with paraben

Type of food	Frequency of consumption			
	Never	1-3 Day/week	4-6 Day/week	Everyday
**Ready-to-drink fruit juices**				
A	98 (75.4)	26 (20.0)	6 (4.6)	0(0.0)
B	94 (72.3)	31(23.8)	5 (3.8)	0(0.0)
C	90 (69.2)	33 (25.4)	5 (3.8)	2 (1.5)
D	107 (82.3)	17 (13.1)	5 (3.8)	1 (0.8)
**Soft Drink**				
A	118 (90.8)	11 (8.5)	1 (0.8)	0(0.0)
B	116 (89.2)	12 (9.2)	2 (1.5)	0(0.0)
C	94 (72.3)	31 (23.8)	4 (3.1)	1 (0.8)
D	81 (62.3)	41 (31.5)	6 (4.6)	2 (1.5)
**Alcoholic beverage**				
A	99 (76.2)	29 (22.3)	2 (1.5)	0 (0.0)
B	79 (60.8)	43 (33.1)	7 (5.4)	1 (0.8)
**Energy drink**				
A	119 (91.5)	10 (7.7)	0(0.0)	1 (0.8)
B	128 (98.5)	2 (1.5)	0(0.0)	0(0.0)
C	127 (97.7)	2 (1.5)	0(0.0)	1(0.8)
D	127 (97.7)	2 (1.5)	0(0.0)	1(0.8)
E	129 (99.2)	1 (0.8)	0(0.0)	0(0.0)
F	128 (98.5)	2 (1.5)	0(0.0)	0(0.0)
**Ready-to-drink canned coffee**				
A	115 (88.4)	12 (9.3)	2 (1.6)	1 (0.8)
**UHT milk**				
A	68 (52.3)	46 (35.4)	10 (7.7)	6 (4.6)
B	95 (73.1)	29 (22.3)	4 (3.1)	2 (1.5)
C	104 (80.0)	18 (13.8)	7 (5.4)	1 (0.8)
D	102 (75.8)	25 (19.2)	3 (2.3)	0 (0.0)
E	85 (65.4)	38 (29.2)	6 (4.6)	1 (0.8)
F	64 (49.2)	41 (31.5)	22 (16.9)	3 (2.3)
**Condensed milk**				

Type of food	Frequency of consumption			
	Never	1-3 Day/week	4-6 Day/week	Everyday
A	122 (93.8)	7 (5.4)	1(0.8)	0 (0.0)
B	110 (84.6)	17 (13.1)	1 (0.8)	2 (1.5)
C	109 (83.8)	17 (13.1)	2 (1.5)	2 (1.5)
D	101 (77.7)	25 (19.2)	3 (2.3)	1 (0.8)
E	96 (73.6)	30 (23.3)	3 (2.3)	1 (0.8)
**Cooking Oil**				
A	54 (41.1)	47 (36.4)	17 (13.2)	12 (9.3)
**Canned fish**				
A	124 (95.4)	6 (4.6)	0(0.0)	0(0.0)
B	87 (66.9)	40 (30.8)	2 (1.5)	1 (0.8)
**Potato chips**				
A	34 (26.2)	83 (63.8)	10 (7.7)	3 (2.3)
**Tomato sauce**				
A	77 (59.2)	42 (32.3)	8 (6.2)	3 (2.3)
**Mayonnaise**				
A	115 (88.5)	14 (10.8)	1 (0.8)	0(0.0)

S5 Table. Descriptive Statistics for dietary paraben intake

Statistics

		Total paraben of Fruit juice	Total paraben of Soft Drink	Total paraben of Alcohol Beverage	Total Paraben of Energy Drink	Total paraben of Canned Coffee	Total paraben of UHT Milk	Total paraben of condensed milk	Total paraben of cooking oil	Total parabe n of canned fish	Total paraben of potato chips	Total paraben of tomato sauce	Total paraben of mayonnaise	Total paraben for this study
N	Valid	130	130	130	130	130	130	130	130	130	130	130	130	130
	Missing	0	0	0	0	0	0	0	0	0	0	0	0	0
Mean		7.2834	7.3911	7.2351	.6829	.258	5.3497	1.1459	.0830	.0954	.1719	.0153	.0071	29.7184
Median		.0000	.0000	.0000	.0000	.000	4.1425	.0000	.0000	.0000	.2390	.0000	.0000	25.1180
Std. Deviation		9.47486	10.85959	10.45802	2.64878	.9005	6.66948	2.27369	.12328	.14543	.15232	.02527	.02054	27.69214
Skewness		1.295	1.636	1.755	4.754	5.074	3.208	3.929	1.858	1.779	1.507	2.414	2.561	1.297
Std. Error of Skewness		.212	.212	.212	.212	.212	.212	.212	.212	.212	.212	.212	.212	.212
Kurtosis		1.109	2.765	5.726	23.719	34.055	15.716	24.607	2.824	5.208	6.286	8.310	4.628	2.155
Std. Error of Kurtosis		.422	.422	.422	.422	.422	.422	.422	.422	.422	.422	.422	.422	.422
Minimum		.00	.00	.00	.00	.0	.00	.00	.00	.00	.00	.00	.00	.00
Maximum		42.23	51.94	65.60	17.82	7.6	45.02	18.35	.43	.87	.84	.13	.07	149.47
Percentiles	50	.0000	.0000	.0000	.0000	.000	4.1425	.0000	.0000	.0000	.2390	.0000	.0000	25.1180
	75	12.6858	12.0803	17.4900	.0000	.000	7.8120	1.7413	.1210	.2490	.2390	.0380	.0000	44.1838

Note: Unit of total paraben is $\mu g/kg\ bw/day$

S6 Table. The consumption patterns of paraben-related food items

Food Related Variables	n (%)	
	Never	Ever
Fruit Juice	56 (43.1)	74 (56.9)
Soft Drinks	74 (56.9)	56 (43.1)
Alcohol Beverages	72 (55.4)	58 (44.6)
Energy Drinks	117 (90.0)	13 (10.3)
Canned Coffee	115 (88.4)	15 (11.6)
Canned Fish	83 (63.8)	47 (36.2)
UHT Milk	115 (88.4)	15 (11.6)
Condensed Milk	82 (62.8)	48 (37.2)
Cooking Oil	54 (41.1)	76 (58.9)
Potato Clips	34 (26.2)	96 (73.8)
Tomato Sause	77 (59.2)	53 (40.8)
Mayonnaise	115 (88.4)	15 (11.6)
